# Clinical implications of apolipoprotein L1 testing in patients with focal segmental glomerulosclerosis: a review of diagnostic and prognostic implications

**DOI:** 10.1097/MS9.0000000000003051

**Published:** 2025-03-03

**Authors:** Aiman Waheed, Muhammad Hamza Gul, Risha Naeem, Sardar Noman Qayyum, Khizra Batool, Abeeha Shaukat, Nashmiya Khan, Safa Irfan Shah, Aisha Rehman Siddiqui, Asad Ullah Farooq, Eeshah Nasir, Samim Noori

**Affiliations:** a Rawalpindi Medical College, Rawalpindi, Pakistan; b Hayatabad Medical Complex, Hayatabad, Pakistan; c Ameer ud din Medical College, Lahore, Pakistan; d Bacha Khan Medical College, Mardan, Pakistan; e Khyber Girls Medical College, Peshawar, Pakistan; f Akhtar Saeed Medical and Dental College, Lahore, Pakistan; g Karachi Medical and Dental College, Karachi, Pakistan; h Wayne State University, Detroit, Michigan, USA; i King Edward Medical University, Lahore, Pakistan; j Ziauddin Medical University, Karachi, Pakistan; k Faculty of Medicine, Nangarhar, Nangarhar University, Nangarhar, Afghanistan

**Keywords:** APOL1, apolipoprotein L1, focal segmental glomerulosclerosis, FSGS, prognostic factor

## Abstract

**Introduction:**

Focal segmental glomerulosclerosis (FSGS) is a leading cause of nephrotic syndrome, contributing to 40% of adult and 20% of pediatric cases globally. Apolipoprotein L1 (APOL1) genetic variants, particularly G1 and G2 alleles, play a pivotal role in FSGS pathogenesis, particularly among African-Americans, where 30–40% carry these risk alleles. These variants impair APOL1 function, causing podocyte injury, proteinuria, and progressive kidney damage. Secondary triggers like infections exacerbate susceptibility. Advances in gene-editing technologies, including CRISPR, offer hope for targeted therapies in FSGS management.

**Objectives:**

This review explores the link between APOL1 variants and FSGS pathogenesis, focusing on their role in podocyte injury and assessing the utility of APOL1 genetic testing in diagnosis and treatment strategies.

**Methodology:**

A systematic literature review was conducted using Medline, PubMed, Google Scholar, and PsychINFO up to April 2024. Of 331 identified articles, 29 relevant studies were analyzed, emphasizing APOL1 variants’ role in FSGS and implications for genetic testing.

**Results:**

About 13% of African-Americans carry APOL1 risk alleles, with 30% having at least one allele. Two risk alleles increase lifetime FSGS risk to 4% and ESKD risk to 7–8%. APOL1-associated kidney damage primarily affects podocytes, accelerating glomerulosclerosis. Emerging treatments, such as inaxaplin, reduced proteinuria by 47%, with 40% achieving remission in FSGS cases linked to APOL1.

## Introduction and background

Focal segmental glomerulosclerosis (FSGS) poses a significant challenge in nephrology, contributing to approximately 40% of nephrotic syndrome cases in adults and 20% in children globally^[1,2]^. This condition is characterized by scarring of the glomeruli – the kidney’s filtration units – resulting in proteinuria (excess protein in urine), hypoalbuminemia, and a progressive decline in kidney function^[3]^. While the etiology of FSGS is multifactorial, encompassing genetic, environmental, and secondary causes, recent research highlights the critical role of apolipoprotein L1 (APOL1) genetic variants, particularly among individuals of African descent. In African-American populations, over 30–40% carry one or more APOL1 risk alleles (G1 or G2), which significantly predispose them to developing FSGS^[3–5]^. The APOL1 gene encodes a small circulating protein involved in innate immunity and lipid transport^[6]^. While the G1 and G2 alleles confer protection against African trypanosomiasis, they simultaneously increase susceptibility to kidney diseases like FSGS and HIV-associated nephropathy (HIVAN), with a 7–10 fold higher risk in homozygotes^[5–7]^. These risk variants are distinguished by specific missense mutations (G1) and a deletion mutation (G2) that disrupt APOL1’s normal cellular function, particularly in podocytes – specialized cells essential for maintaining the glomerular filtration barrier^[4]^.

**HIGHLIGHTS**

- **APOL1 Risk Variants and FSGS Progression**: The review emphasizes the critical role of apolipoprotein L1 (APOL1) risk variants in the development and progression of focal segmental glomerulosclerosis (FSGS), particularly among individuals of African descent.
- **Diagnostic and Prognostic Value**: It highlights how genetic screening for APOL1 variants can enhance diagnostic accuracy and serve as a prognostic tool for managing FSGS in high-risk populations.
- **Mechanisms of Podocyte Injury**: The manuscript delves into the molecular mechanisms through which APOL1 variants induce podocyte damage, leading to FSGS, and discusses the pathophysiology of the disease.
- **APOL1-Targeted Therapies**: It explores the potential of APOL1-targeted therapies, which show promise in improving the outcomes of patients with APOL1-associated FSGS.
- **Clinical Integration**: The review suggests that integrating APOL1 genetic testing into routine clinical practice could lead to personalized treatment strategies and better patient management in nephrology.

Mechanistically, APOL1 risk alleles alter intracellular trafficking, leading to cytotoxicity and podocyte damage through lysosomal dysfunction, impaired mitochondrial activity, and disrupted autophagy. Podocyte apoptosis and detachment from the glomerular basement membrane progressively weaken the filtration barrier, exacerbating proteinuria and kidney damage^[5,7]^. It is important to note that possessing APOL1 risk variants does not guarantee FSGS development. Environmental triggers, such as viral infections or second hits (e.g., suPAR), interact with genetic susceptibility, accelerating disease onset^[8]^. For instance, studies report that around 70% of individuals with both APOL1 risk alleles develop FSGS or related nephropathies only in the presence of additional stressors^[4,7]^. The discovery of APOL1’s involvement in FSGS has spurred significant therapeutic interest. Current strategies focus on silencing APOL1 expression, preventing its cytotoxic effects, or genetically editing the mutant alleles through technologies like CRISPR-Cas9. For example, gene-silencing therapies in preclinical studies have demonstrated a 50–70% reduction in podocyte injury^[8,9]^. Although challenges persist, such as safety concerns surrounding gene therapy, these advances hold promise for improving outcomes in high-risk populations. The present state of APOL1 and FSGS research is encouraging despite these challenges. Through deciphering the intricate mechanisms of podocyte damage and exploring diverse therapeutic strategies, researchers are preparing the way for a future in which individuals with APOL1 risk variants may benefit from expanded therapeutic options and potentially even prevent the onset of FSGS.

## Methodology

### Literature search

A comprehensive literature search was conducted on apolipoprotein L1 (APOL1) and focal segmental glomerulosclerosis (FSGS) across multiple databases, including PubMed, Google Scholar, Medline, and PsychINFO, up to April 2024. Specific search terms such as “apolipoprotein L1,” “focal segmental glomerulosclerosis (FSGS),” “nephrotic syndrome,” and “prognostic factor” were used to identify relevant studies.

### Study selection

The first author independently reviewed all articles retrieved from the search. Titles, abstracts, and reference lists were screened by the first and second authors against predefined eligibility criteria. To ensure a systematic approach, studies were assessed for inclusion based on the PRISMA framework.

### Eligibility criteria

Studies were included based on predefined PICO criteria:

- (P) Population: Patients diagnosed with FSGS.
- Intervention or Exposure: Related to APOL1.
- (C) Comparison: Standard or comparator groups.
- (O) Outcomes: Relevant to FSGS progression or prognosis.

Eligible studies included systematic reviews and review articles focusing on APOL1 and FSGS. Clinical trial registers and randomized controlled trials (RCTs) were not included in the search. Only English-language studies were considered. Articles unrelated to FSGS or APOL1, non-English studies, and duplicates were excluded.

### Data extraction

Data extraction was performed independently by the authors using standardized techniques, including Excel spreadsheets for data organization and EndNote for managing references. Extracted data included study characteristics (author, year, and design), patient demographics (age, sex, and ethnicity), intervention details (APOL1 variants and treatments), results (outcomes and prognosis), and conclusions. Discrepancies in data extraction were resolved through team discussions to ensure accuracy and consistency.

**Figure 1.**
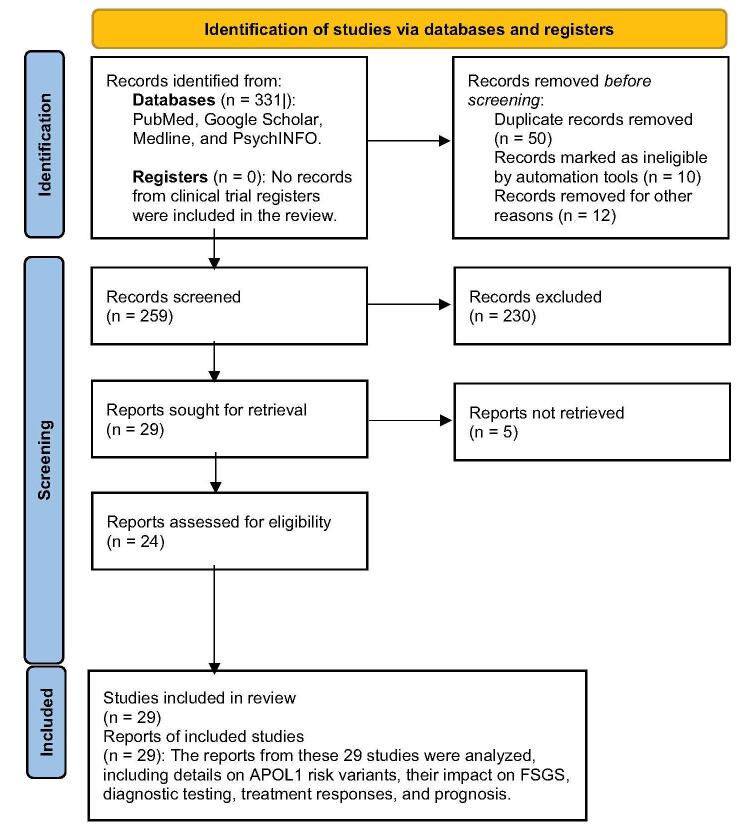
PRISMA flow diagram for the selection of studies on apolipoprotein L1 and focal segmental glomerulosclerosis (FSGS).

The study selection process, including identification, screening, eligibility assessment, and final inclusion of studies, is detailed in the PRISMA flowchart of Fig. 1.

## Results

Through a comprehensive literature search, we identified 331 published articles, of which 29 met our inclusion criteria and were included in this narrative review. We have explored the APOL1 risk variants, their prevalence and demographics, and clinical implications.

### High-risk APOL1 genotypes

High-risk APOL1 genotypes, G1 and G2, are significantly associated with focal segmental glomerulosclerosis (FSGS) and chronic kidney disease (CKD), particularly in individuals of African ancestry. Approximately 13–15% of African Americans carry at least one high-risk genotype, with 1–2% carrying two risk alleles. The G1 variant involves amino acid substitutions (S342G and I384M), while the G2 variant is characterized by a six-base pair deletion, resulting in the loss of two amino acids. These mutations impair APOL1 function, disrupting podocyte homeostasis and leading to kidney damage. These variants are nearly absent in non-African populations, highlighting their evolutionary origin in response to *Trypanosoma brucei* infections in sub-Saharan Africa.

### Mechanisms of kidney injury

**Figure 2.**
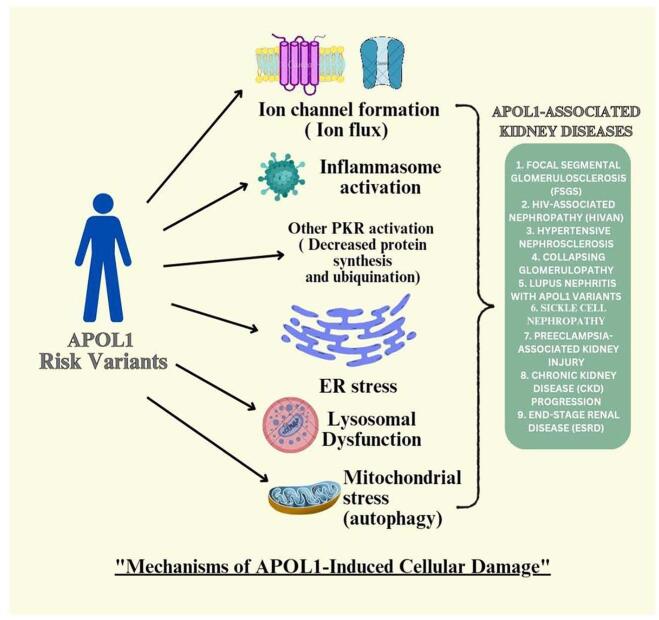
APOL1-induced cellular damage mechanism.

APOL1 variants disrupt intracellular trafficking and cytoskeletal integrity in podocytes, leading to cellular stress, apoptosis, and glomerular filtration defects. Additionally, APOL1 affects endothelial cells, immune cells (T cells and natural killer cells), and vascular smooth muscle cells, causing systemic renal pathology and exacerbating CKD. The mechanism of cellular injury due to APOL1 risk variants is shown in Fig. 2.

### Role of circulating permeability factors (CPFs)

CPFs contribute to podocyte dysfunction and proteinuria, which accelerate the progression of FSGS. These factors interact with high-risk APOL1 genotypes to amplify disease severity.

### Mechanistic insights

APOL1 genetic testing identifies individuals at risk of developing kidney disease, particularly among African-Americans. Testing is increasingly utilized to predict disease progression and guide tailored therapeutic strategies. High-risk genotyping also offers a prognostic value by predicting treatment responses in FSGS and CKD patients.

### Emerging therapeutic approaches

Therapeutic advancements include the development of APOL1 inhibitors aimed at mitigating the harmful effects of high-risk variants. Gene-editing approaches, such as CRISPR-Cas9, hold potential for correcting these mutations, offering curative solutions. Trials targeting APOL1 pathways are ongoing, aiming to address the unmet needs of high-risk populations.

Table 1 indicates the summary findings of the narrative review.

**Table 1:** Summary of findings from the narrative review

Author	Year	Study type	Aspect	Findings	Reference
Friedman DJ et al.	2021	Review	APOL1 variants	High-risk APOL1 genotypes (G1 and G2) are strongly associated with an increased risk of developing focal segmental glomerulosclerosis (FSGS), particularly in individuals of African descent.	^[6]^
Friedman DJ et al.	2021	Review	High-risk genotypes	The G1 genotype involves specific amino acid substitutions, while G2 is characterized by amino acid deletions. These mutations disrupt normal APOL1 function, leading to kidney damage.	^[6]^
Pollak MR et al.	2023	Review	Mechanisms of podocyte injury	APOL1 risk variants may disrupt trafficking within podocytes, causing cellular stress and death. Additionally, impaired interactions with the glomerular basement membrane weaken filtration.	^[22]^
Friedman DJ et al.	2021	Review	Impact on non-podocyte cells	APOL1 risk variants also affect endothelial cells, T-cells, natural killer cells, and vascular smooth muscle cells, influencing broader renal pathology.	^[6]^
Egbuna O et al.	2023	Clinical Trial	Circulating permeability factors in FSGS	Circulating permeability factors (CPFs) contribute to podocyte dysfunction and proteinuria, exacerbating FSGS.	^[24]^
Cruz NM et al.	2018	Review	Therapeutic interventions	Emerging therapies include APOL1 inhibitors and gene editing approaches (e.g. CRISPR-Cas9) targeting high-risk APOL1 variants.	^[9]^
Friedman DJ et al.	2021	Review	Prognostic value of APOL1 genotyping	APOL1 genetic testing identifies high-risk individuals and predicts treatment response, aiding tailored management strategies.	^[6]^

## Discussion

### Apolipoproteins: role in kidney function and disease

The APOL gene family consists of six members located on human chromosome 22. APOL1 is one such member, which is expressed in kidney endothelial cells, podocytes, epithelium of proximal convoluted tubules, renal arteries, and renal arterioles. G0 is a nonrisk variant, while G1 and G2 are risk variant alleles that arose due to amino acid deletions. The high-risk genotypes of APOL1 include two risk alleles in any combination, for example, G1/G1, G2/G2, or G1/G2. The odds ratio for high-risk genotypes is high for FSGS compared to individuals carrying low-risk genotypes. APOL1 risk variants may create pores in the kidney cell membranes or lead to mitochondrial dysfunction and injury – two of the hypotheses proposed^[6]^. The density of the APOL1 risk variant has increased in African populations during the past 10,000 years, wherein a single G1 or G2 allele provided immunity against the African sleeping sickness parasite *Trypanosoma brucei* rhodesiense. Although protection against this type of African sleeping sickness through APOL1 risk variants is no longer relevant, allele frequency remains high^[10]^. APOL1 is inherited as a recessive trait, while G1 and G2 variants lead to malignant, gain-of-function effects on the encoded protein^[11]^. Most people with high-risk APOL1 genotypes do not develop overt kidney disease, which suggests that they either possess a protective factor and/or vulnerability to other components that may be needed for the disease to develop, often referred to as “second hits.” Therefore, it is hard to assign any kidney disease to APOL1 alone^[12]^.

### Focal segmental glomerulosclerosis (FSGS): insights and pathophysiology

The characteristic feature of FSGS is the gradual formation of scar tissue within the glomeruli^[13]^. This scarring process primarily affects podocytes, which play a crucial role in kidney function. Clinically, FSGS presents with significant proteinuria. Other standard features include hypoalbuminemia, generalized edema, and elevated cholesterol levels^[14]^. Focal segmental glomerulosclerosis is classified as primary (idiopathic) when no cause is identified and secondary when the underlying etiology is known^[2]^. The improvement in renal function following plasmapheresis suggests that podocyte injury in FSGS results from circulating permeability factors. Proteinuria arises from enhanced albumin permeability of chloride channels-1 (CLC-1), which is controlled by CRLF1, JAK2, and STAT3 gene inhibitors^[15]^. The soluble urokinase-type plasminogen activator receptor (SuPAR) reduces podocyte function and is associated with proteinuria^[16]^. It can be treated with urokinase-type plasminogen activator receptor (uPAR)-specific antibodies. Despite being more closely linked to minimal change disease (MCD), angiopoietin-like 4 protein (Angptl4) contributes to the pathophysiology of FSGS through hypertriglyceridemia.

FSGS can occur at any age, with 7–20% of children and 40% of adult idiopathic nephrotic syndrome cases attributed to it^[17]^. The risk is 1.5 times higher in men than in women. Black patients face a four-fold increased risk of end-stage renal disease (ESRD) compared to Caucasian and Asian patients^[18]^. The genetic basis of FSGS may arise from familial inheritance patterns, including X-linked, autosomal dominant, autosomal recessive, or mitochondrial patterns, or it may occur randomly^[19]^. FSGS, especially when familial, is associated with genetic changes affecting α-actinin-4 (ACTN4), transient receptor potential cation channel-6 (TRPC6), and CD2-related protein (CD2AP). These mutations can affect the form and function of podocytes, which are critical glomerular filtration barrier cells. These genetic changes that affect podocyte function can lead to hallmark signs of FSGS, such as proteinuria and progressive glomerulosclerosis.

### Relationship between APOL1 genotype and focal segmental glomerulosclerosis (FSGS)

Ongoing research is exploring the complex relationship between the APOL1 genotype and FSGS. Within FSGS, collapsing glomerulopathy (CG) presents distinctive morphological features. Studies have shown a notable association between African-American individuals diagnosed with CG, particularly those treated with interferon therapy, and the presence of two APOL1 risk variants. Moreover, cases have been documented where African-American patients with lupus nephritis and two APOL1 risk variants develop collapsing lesions. These insights emphasize the need for continued investigation into the role of the APOL1 genotype in the pathogenesis of CG, opening up potential avenues for understanding and managing this condition^[20]^. The clinical implications of APOL1 in FSGS are summarized in Table 2.

**Table 2:** Clinical implications of APOL1 testing in focal segmental

Authors	Year	Study Type	Study Design	Methods	Main Findings	Ref. No.
Friedman & Pollak	2021	Review	Comprehensive review	Overview of APOL1 nephropathy and its implications in FSGS	Highlights diagnostic and prognostic value of APOL1 testing in FSGS patients.	^[6]^
De Vriese et al.	2021	Review	Comprehensive review	Review of APOL1 testing and therapeutic strategies	Discusses how APOL1 testing informs prognosis and guides treatment in FSGS.	^[26]^
Dummer et al.	2015	Review	Comprehensive review	Overview of APOL1 risk variants in kidney disease	Explores the role of APOL1 variants in FSGS diagnosis and prognosis.	^[12]^
Egbuna et al.	2023	Clinical Trial	Randomized controlled trial	Evaluated inaxaplin’s effect in APOL1 variant carriers	Inaxaplin’s impact on proteinuria in APOL1-positive FSGS patients, indicating potential therapeutic benefit.	^[25]^
Salfi et al.	2023	Review	Comprehensive review	Review of molecular mechanisms and therapeutic targets	Provides insights into APOL1 nephropathy mechanisms relevant for diagnostic and prognostic assessments in FSGS.	^[29]^

### Interconnection between APOL1 variants and FSGS

Variants in the APOL1 gene are closely linked to FSGS, a kidney disease that is common among people who are African-American. Significant proteinuria is a hallmark of this genetic link, which causes ESRD in individuals of sub-Saharan African heritage. It also speeds up the disease’s progression. Studies on 94 FSGS patients with nephrotic syndrome revealed that 27 of them had these high-risk gene variants. Interestingly, at the time of diagnosis, these patients were typically older and had lower baseline kidney function^[20]^.

### APOL1 variants, p38 signaling, and hyperexpression in FSGS

From studies conducted by Olabisi *et al*., it has been demonstrated that specific variants of the APOL1 gene are associated with hyperexpression of the p38 MAPK pathway in patients with FSGS. This hyperexpression occurs prominently in intrinsic renal cells such as podocytes, endothelial cells, and tubular cells, as well as in infiltrating macrophages, neutrophils, and alpha smooth muscle actin (α-SMA) positive myofibroblasts. The activation of p38 signaling pathways due to APOL1 variants subsequently leads to dysregulation of the podocyte cytoskeleton. This mechanistic insight suggests that APOL1-induced hyperactivation of p38 signaling may play a crucial role in the pathogenesis of FSGS, particularly in populations of African ancestry who are more likely to carry these genetic variants^[1]^. Table 3 shows the overview of APOL1 variants and their association with kidney disease.

**Table 3:** Overview of APOL1 variants and their association with kidney diseases

Authors	Year	Study type	Study design	Methods	Main findings	Ref. No.
Kopp et al.	2015	Case-Control	Retrospective cohort analysis	Genotyping APOL1 variants; clinical assessment of kidney function	APOL1 risk alleles significantly associated with increased risk of FSGS.	^[4]^
Limou et al.	2014	Population-based	Cross-sectional study	Genotyping; population-based survey of kidney health	APOL1 variants prevalent in African populations; associated with higher risk of chronic kidney disease.	^[5]^
Friedman & Pollak	2021	Experimental	Review and meta-analysis	Review of genetic and clinical data on APOL1 nephropathy	APOL1 variants linked to significant kidney damage and disease progression.	^[6]^
De Vriese et al.	2018	Longitudinal	Prospective cohort study	Long-term follow-up; genotyping; clinical outcomes monitoring	APOL1 risk variants linked to accelerated progression of kidney disease.	^[19]^

### Predictive biomarkers and pathogenic pathways in FSGS involving APOL1

The goal of recent studies on APOL1 variant-related FSGS has been to clarify pathogenic pathways and identify predictive biomarkers. Additionally, studies have revealed increased tumor necrosis factor-alpha (TNF-α) and its receptor levels in the plasma and urine of FSGS patients, highlighting the disease’s inflammatory aspect and possible treatment targets. Clinical findings indicate that therapies targeting TNFα, such as infliximab and etanercept, have reduced proteinuria in recurrent FSGS patients. These results point to a complex interplay in APOL1-associated FSGS between genetic variants, biomarkers, and pathogenic pathways, which paves the way for targeted therapeutic intervention^[21]^.

### Role of APOL1 genetic testing in diagnosing FSGS

Not everyone with the high-risk APOL1 genotype develops renal disease, suggesting that in susceptible individuals, other factors – referred to as “second hits” might be required to trigger APOL1-related kidney diseases. High-risk APOL1 genotypes have a substantial impact on an individual’s susceptibility to developing FSGS, even though they may not always manifest overt kidney disease. Genetic testing for APOL1 variants can help with early diagnosis, treatment planning, and, ultimately, patient outcomes. Early identification of individuals with high-risk genotypes enables healthcare providers to manage and potentially postpone the onset of FSGS-related complications^[22]^. APOL1 genetic testing may benefit anyone presenting with typical renal syndromes, regardless of race, as APOL1 risk alleles can occur in people other than African descent. The management and prognosis of people with high-risk APOL1 genotypes are frequently complicated by the presence of extensive fibrosis and more severe kidney damage at diagnosis. These cases highlight the urgent need for specialized therapeutic approaches that address the underlying genetic predisposition, given the quick progression to end-stage renal disease. Therefore, genetic testing is impertinent to initiate intensive care earlier for patients with two risk alleles diagnosed with FSGS^[4]^.

### Prognostic value of APOL1 variants in FSGS

- ***Impact of APOL1 variants on disease progression***:

Individuals have 20 times more probability of progressing to FSGS with high-risk APOL1 genotypes. FSGS is the most common type of APOL1-associated nephropathy^[22]^. In the presence of high-risk alleles, with age and overall kidney health, risks for developing FSGS vary. The existence of APOL1 risk alleles constitutes a higher risk of kidney diseases, particularly FSGS, in the younger population compared to older individuals, where overall rare nephropathies are lower^[23]^.

APOL1 risk genotypes in Black individuals affect kidneys at an early age and cause FSGS. The disease is likely to be more severe in the setting of proteinuria in Black children with APOL1 risk variants, with lower estimated glomerular filtration rates (eGFR) at diagnosis and faster yearly eGFR declines. CG is a type of focal segmental glomerulosclerosis and is distinguished by parietal epithelial cell hyperplasia due to podocyte progenitor damage and is often associated with endothelial tubulo-reticular inclusions seen on electron microscopy in certain conditions such as hemophagocytic lymphohistiocytosis, systemic lupus erythematosus, HIV, parvovirus B19 infection, etc. In African-Americans with two APOL1 risk alleles, CG frequently co-exists with nephrotic-range proteinuria and acute kidney injury (AKI). While African-Americans are more prone to the collapsing form of FSGS in HIV-positive patients, the severity of pathologic lesions in HIV-associated nephropathy is comparable in patients with and without APOL1 risk alleles, suggesting other factors influencing the severity and phenotype of FSGS in these patients^[24]^.

- ***APOL1 variants and response to therapy*:**

Recent studies suggest that individuals with high-risk alleles, especially those of African-American descent, may react differently to angiotensin-converting enzyme inhibitors (ACEIs) and other hypertensive medications, reducing blood pressure more as compared to individuals without the genotype. Although harmful in its gain-of-function variations, apolipoprotein L1 is generally not essential for the normal functioning of the kidneys, in contrast to the proteins necessary for kidney function. Thus, reducing APOL1 activity may be less difficult than blocking critical proteins, and since people without functional APOL1 do not exhibit any symptoms of renal disease, it is unlikely that blocking the protein will cause kidney dysfunction. This explains the treatment of FSGS in individuals with APOL1 mutations^[23]^.

Glucocorticoids and other medications that address suspected immunologic abnormalities are usually administered along with diuretics and renin-angiotensin-aldosterone system inhibitors. However, these treatments have significant adverse effects and limited efficacy. Due to the pathogenic mechanism of harmful gain-of-function APOL1 mutations causing glomerular injury, researchers postulated that blocking the activity of the APOL1 channel could significantly lessen the damage to podocytes and proteinuria, thereby delaying progressive kidney disease. This hypothesis has led to promising developments including inaxaplin, an oral, selective, small-molecule blocker of APOL1 channel activity. In a phase two clinical trial, inaxaplin significantly reduced proteinuria in people with FSGS confirmed by biopsy and two mutations in APOL1. Preclinical research revealed that inaxaplin decreased proteinuria in a mouse model and particularly inhibited APOL1 channel activity in vitro. In the research study, the mean reduction in the urine protein-to-creatinine ratio over 13 weeks was 47.6% for 13 persons treated with inaxaplin who met adherence standards; similar reductions were seen in practically all subjects^[25]^.

The capacity of the G1 and G2 mutations that cause APOL1 nephropathy to harm the kidneys without being necessary for kidney development or function makes them special. According to this, blocking the faulty APOL1 gene could mitigate the disease without having a major negative impact, although it might make people more susceptible to trypanosomiasis. Promising results have been shown in studies engaging the antisense oligonucleotides to silence the aberrant gene in APOL1-transgenic mice, therefore lowering kidney damage and proteinuria. In patients with FSGS lesions and proven APOL1 G1/G1, G2/G2, or G1/G2 genotype, a clinical trial called NCT04340362 is now being conducted to assess the effectiveness, safety profile, and pharmacokinetics of an oral APOL1 inhibitor called VX-147. Even with these developments, the majority of genetic variants of FSGS remain resistant to immunosuppressive treatments such as alkylating drugs and steroids. Genetic testing for APOL1 risk variants can significantly impact treatment outcomes by identifying patients who might benefit from targeted therapies, including novel APOL1 inhibitors and tailored immunosuppressive treatments. Incorporating APOL1 genetic testing into clinical practice enhances the optimization of therapeutic strategies for FSGS, which may lead to improved long-term renal outcomes and facilitate personalized treatment approaches based on specific APOL1 risk variants^[26]^. Moreover, APOL1-associated FSGS is associated with quick development to end-stage renal disease and significant kidney fibrosis in people of different ancestries. Although aggressive initial treatment options could be guided by genetic testing for APOL1, the evidence now available does not support the idea that intensive treatment with currently available medications significantly slows the course of the disease in high-risk patients. A deeper comprehension of APOL1-induced glomerular and microvascular damage is necessary, given the continuous pathologic processes mediated by APOL1 variant proteins. This knowledge is essential for creating more potent medicines that can overcome the present medication’s limited efficacy and provide hope for better management of APOL1-associated nephropathies^[4]^.

### Long-term outcomes for patients with APOL1-associated FSGS

APOL1-associated kidney disease patients frequently exhibit severe vascular and interstitial abnormalities along with a range of lesions that vary from glomerulosclerosis to focal segmental glomerulosclerosis^[27]^.

Hypertension is a usual comorbidity in individuals with APOL1-associated FSGS, particularly African-Americans, often developing before a decline in renal function. This hypertension is more severe and may cause other cardiovascular problems, which could worsen the situation in the long run. Researchers used information from the Mount Sinai BioMe Biobank to examine African-Americans’ blood pressure about their APOL1 gene variations. In addition to adding participants from additional biobanks – 1,623 from BioMe, 1,809 from Vanderbilt BioVU, and 567 from Northwestern NUgene – they included 5,200 African-American participants. According to their findings, 14–16% of African-Americans with two dangerous variations of the APOL1 gene had higher blood pressure and were diagnosed with hypertension earlier in life. In particular, people between the ages of 20 and 29 and every dangerous variant of APOL1 elevated the systolic blood pressure by 0.94 mmHg. A 10-year reduction in kidney function was also seen in these young people by the time they were thirty to forty-nine years old. This implies that the reduction in kidney function may have been caused by or preceded by high blood pressure^[28]^.

## Limitations and gaps

The reviewed studies highlight several limitations and gaps in our understanding of APOL1-related kidney diseases. One major limitation is the lack of population diversity, as most studies primarily focus on African-American or sub-Saharan African populations. This limits the generalizability of findings to other ethnic groups, leaving a significant gap in understanding how APOL1 risk variants might affect non-African populations. Furthermore, the variability in study designs, including cohort and case-control studies, complicates cross-study comparisons and reduces the clarity of results. Many studies also lack long-term follow-up data, with only a few longitudinal studies tracking the progression of kidney disease in APOL1 carriers over extended periods. This absence of long-term data makes it difficult to predict the full impact of APOL1 risk variants on renal health, especially in terms of long-term outcomes such as end-stage renal disease (ESRD). Additionally, while it is well-established that second hits such as hypertension and diabetes exacerbate kidney damage in APOL1 carriers, the specific environmental or genetic triggers for these second hits remain largely undefined, which limits the understanding of their role in disease progression. Last, there is a significant gap in the development of therapeutic strategies aimed at targeting the APOL1-related pathophysiology. While some studies focus on managing symptoms like proteinuria and hypertension, few studies address how to intervene directly with the genetic mutations that cause kidney disease in APOL1 carriers, indicating the need for more focused research into personalized treatments for these individuals.

### Interpretation of findings

The interpretation of findings suggests a strong link between APOL1 genetic variants and the pathogenesis of focal segmental glomerulosclerosis (FSGS). The high-risk genotypes of APOL1, specifically the G1 and G2 variants, are associated with increased susceptibility to kidney damage, particularly in individuals of African descent. These genetic variants contribute to the development of FSGS by inducing podocyte dysfunction, mitochondrial injury, and activation of harmful cellular signaling pathways. Despite not all carriers of these variants developing kidney disease, the presence of APOL1 risk alleles significantly raises the risk of progressing to FSGS and other renal diseases, particularly when combined with environmental factors or “second hits.” The findings also highlight the importance of genetic testing for APOL1 variants, as it can aid in early diagnosis and inform personalized treatment strategies, potentially improving patient outcomes. Furthermore, targeted therapies aimed at inhibiting APOL1 channel activity are emerging as promising treatments to mitigate kidney damage and slow disease progression.

## Conclusion

Apolipoprotein L1, produced by the APOL1 gene on chromosome 22, impacts kidney disease progression, especially in African individuals. The G1 and G2 genetic variants follow a recessive inheritance pattern and increase the risk of FSGS, HIV-related nephropathy, and hypertension-induced CKD. APOL1 inhibitors show promise in treating APOL1-associated FSGS. Genetic screening for APOL1 variants is crucial, especially in African populations, but broader screening is also needed. Integrating genetic testing into clinical practice has the potential for managing APOL1-associated nephropathies. Future efforts should focus on expanding clinical indications, and refining techniques, and treatment to maximize the benefits of APOL1 diagnosis for a spectrum of patients with focal segmental glomerulosclerosis. Future research should focus on expanding the scope of genetic screening to include broader populations, refining diagnostic techniques for improved accessibility and sensitivity, and conducting longitudinal studies to better understand the long-term impacts of APOL1 variants. Additionally, clinical trials assessing the efficacy and safety of APOL1 inhibitors should be prioritized, alongside efforts to develop new treatment strategies and personalized medicine approaches tailored to APOL1-associated kidney diseases. These efforts will help optimize the use of APOL1 genetic testing and improve outcomes for patients with FSGS and related nephropathies.

## Data Availability

The data are available from authors upon request and contain a data extraction sheet and Revman files.
